# Understanding the Impact COVID-19 Has Had on Traumatic Ankle Fractures in a UK Orthopaedic Department: Should Current Guidelines Be Revised?

**DOI:** 10.7759/cureus.73455

**Published:** 2024-11-11

**Authors:** Harnoor Khroud-Dhillon, Alexander Jaques, Tien Yeoh, Akshay Date, Abdel-Rahman Abdel-Fattah, Bhavana Selvarajah, Karanjeev Johal

**Affiliations:** 1 Trauma and Orthopaedics, Lister Hospital, Stevenage, GBR; 2 Orthopaedics, King's College Hospital, London, GBR; 3 General Medicine, Lister Hospital, Stevenage, GBR

**Keywords:** ankle fracture, lockdown, conservative management, covid-19 pandemic, lower limb trauma

## Abstract

Introduction

Ankle fractures are common yet debilitating injuries. Surgery is the mainstay treatment for ankle fractures displaying a high suspicion of instability. During the coronavirus disease 2019 (COVID-19) pandemic, conservative management became prominent in patients with ankle fractures. This study assesses the variation in ankle fracture management during the COVID-19 versus pre-COVID-19 pandemic and also highlights how the lockdown affected patient’s behaviour.

Methods

Data for patients with acute ankle fractures during the COVID-19 pandemic (March-June 2020) were retrieved using an electronic trauma database (eTrauma, Open Medical, London, UK). Patients were categorised as operative versus non-operative. The same data were extrapolated from eTrauma for pre-COVID times (March-June 2019). Data were cross-checked with inpatient notes. Patient demographics, mechanism of injury, patient outcomes (Foot and Ankle Outcome Score (FAOS)) and delay in presentation were also analysed.

Result

There was an increased number of acute ankle fractures referred to the orthopaedic department during the COVID-19 pandemic (n=161, 78.9% increase). However, 52% more cases were managed conservatively when compared with pre-COVID times. We also identified an increased presentation time from the date of injury to the orthopaedic review, along with a non-statistically significant decreased delay to surgery during the COVID-19 pandemic (p=0.131). Compared to 2019, FAOS scores were greater during the COVID-19 pandemic (p=0.049), despite no difference in the American Society of Anesthesiologists (ASA) grade between the two time periods.

Conclusion

Compared to the pre-COVID-19 pandemic period, more ankle fractures were managed conservatively in 2020, despite no significant difference found in baseline morbidity. In addition, patient outcomes were greater in 2020. Based on the data analysed, it would be prudent to review current ankle fracture management guidelines and consider a lower threshold for conservative management.

## Introduction

​The coronavirus disease 2019 (COVID-19) is a contagious respiratory disease that has caused healthcare systems and global economies to severely struggle within the space of a few months. As of October 2020, there have been over 705,000 confirmed cases and over 43,000 deaths within the UK alone [1]. The first reported death attributed to COVID-19 within the UK was on February 28, 2020, and by March 26, 2020, the British Government had implemented a nationwide lockdown in an attempt to reduce infection rates and protect the NHS. This lasted three months with ease of restrictions starting to make an appearance in mid to late June 2020. However, despite the nation being in lockdown for three months, COVID-19 infection rates increased, which led to an increased strain on the NHS. This led to the ubiquitous slogan ‘Stay home, Protect the NHS, Save lives’. Consequently, local NHS trusts took an immediate and pragmatic approach and cancelled many elective surgeries, during the peak of the pandemic and to reserve surgical intervention for absolute emergencies. It was estimated that restrictions on activity, including ‘lockdowns’, were imposed on nearly four billion people worldwide in 90 countries/territories [2]. This restriction on movement is the biggest sudden change to human behaviour for generations, and limited analysis has occurred on the effects of these changes in human behaviour. This study aims to highlight how this approach has impacted ankle fracture management within the orthopaedic department at the Lister Hospital, East and North Hertfordshire NHS Trust and also draws attention to important data pertaining to ankle fractures as a result of the COVID-19 lockdown guidance.

## Materials and methods

Study design

This is a single-centre retrospective cohort study of adult patients with acute traumatic ankle fractures.

Inclusion criteria

​Data were collected on all patients who presented to the orthopaedic department with acute traumatic ankle fractures during the study period from March 1, 2020, to June 30, 2020. This study period was selected to ensure all patients during the lockdown period, implemented by the UK Government, were captured in our study. Similar data pertaining to ankle fractures were collected from March 1, 2019, to June 30, 2019, to act as a comparison. We defined ankle fractures as fractures that involved one or more malleoli (medial, lateral and/or posterior).

Exclusion criteria

​Patients who presented with ankle fractures outside the designated study period, i.e., March 1, 2019/2020, to June 30, 2019/2020, were excluded from this study. Additionally, fractures that were not anatomically considered ‘ankle fractures’ of the hindfoot, midfoot or proximal tibia/fibula were not included in our study. Procedures that were not ‘open reduction and internal fixation’ (ORIF) or ‘application of external fixator’ were also not included in this study.

Patient sampling

​Patient data including date of injury, date and type of surgery (if proposed), management proposed, mechanism of injury and age and gender of the patient were extrapolated using an electronic trauma database (eTrauma, Open Medical, London, UK) [3]. This was then cross-checked with inpatient notes, operation notes, theatre records and discharge summaries, which were all requested from the hospital records department. The total number of cases, days in the delay of presentation and days from referral to operation were calculated using Microsoft Excel (Microsoft Corp., Redmond, WA). Patients who returned to the theatre and/or had multiple referrals during the same admission were recorded only as their first presentation. This was to mitigate the overrepresentation of these data points on the analysis of delay in presentation and operation. Data comparing differences in patient outcomes using Foot and Ankle Outcome Score (FAOS) scores and American Society of Anesthesiologists (ASA) physical status scores between the 2019 and 2020 cohorts were analysed. In addition, differences in patient outcomes by sex and number of days from injury to surgery were analysed separately. All statistical analyses were conducted using SPSS Statistics Software package version 27 (IBM Corp., Armonk, NY). This study was registered and performed in collaboration with the East and North Hertfordshire NHS trust audit department. There was no missing data.

## Results

Management

**Figure 1:**
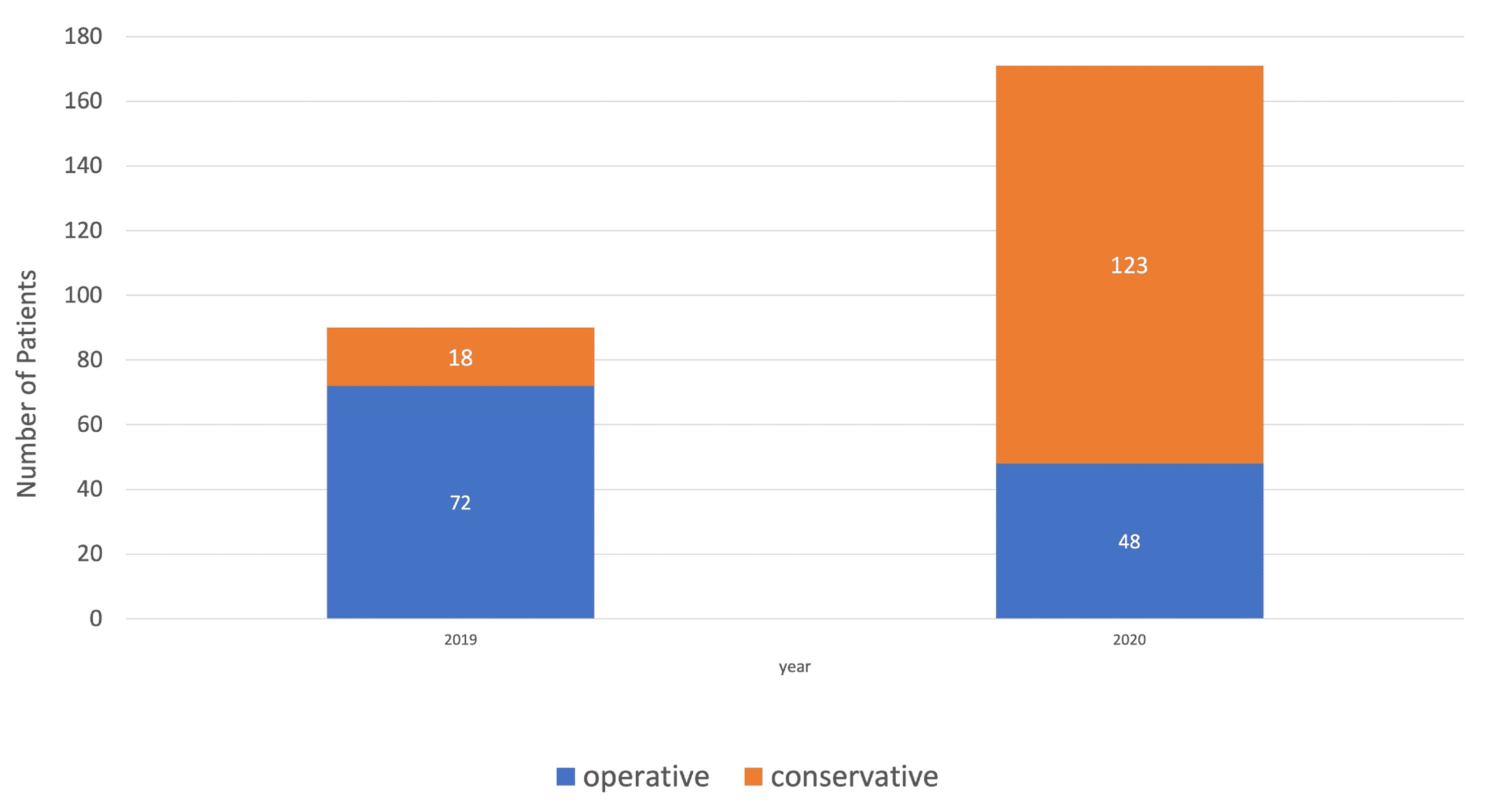
Percentage change in operative versus conservative management for ankle fractures between March and June 2019 and 2020

​During the COVID-19 lockdown phase (March 1, 2020, to June 30, 2020), the orthopaedic department at Lister Hospital saw 161 ankle fracture cases, an increase of 78.9% from 90 cases in March to June 2019. Of these cases, 80% were managed operatively in 2019, a stark contrast to that of 2020 with only 28% of cases being managed operatively (Figure 1). Of the acute ankle fractures in March-June 2019, 20% were managed conservatively compared to 72% in March-June 2020.

Patient demographics

​The average age of patients who presented to the Lister Hospital orthopaedic department with an acute ankle fracture in the study period of 2019 was 49.2 years old. This was comparable to the 2020 average, which was 47.5 years old (p=0.579) (Table 1). The total number of acute ankle injuries from March to June 2019 was 90. Of these cases, 63.3% were female and 36.7% were male. In the 2020 study period, there was a total of 161 cases, of which 58.4% were female and 41.6% were male (Table 1).

**Table 1:** Patient demographics (age and gender) of those who presented with acute ankle fractures in March-June 2019 and 2020

	March to June 2019			March to June 2020		
Age (years)	Female (number of patients)	Male (number of patients)	Total (number of patients)	Female (number of patients)	Male (number of patients)	Total (number of patients)
0-18	6	6	12	11	14	25
19-30	4	8	12	6	9	15
31-50	9	10	19	29	16	45
51-74	27	7	34	33	23	56
>75	11	2	13	15	5	20
Total	57	33	90	94	67	161

​The age group that presented with the highest number of acute ankle injuries in both study periods was the 51-74-year-old group (Table 1). Table 1 illustrates that from March to June 2019, 34 patients (27 female patients and seven male patients) were aged between 51 and 74 years old, which represented 37.8% of the acute ankle fracture cases in the 2019 study period. Similarly, in the 2020 study cohort, 56 patients (33 female patients and 23 male patients) were aged between 51 and 74 years old, which represented 34.9% of this cohort.

​The number of male patients who presented with acute ankle trauma in the 51-74-year-old age group was seven in 2019. In 2020, the numbers increased by more than threefold (n=23). Additionally, male referrals of any age doubled in number from 33 male cases in March-June 2019 to 67 in March-June 2020 (Table 1). In addition, the ASA physical status score did not differ significantly between the 2019 and 2020 cohorts (OR: 2.87, p=0.409 (Fisher’s exact test)).

Delay in presentation and surgery

**Figure 2:**
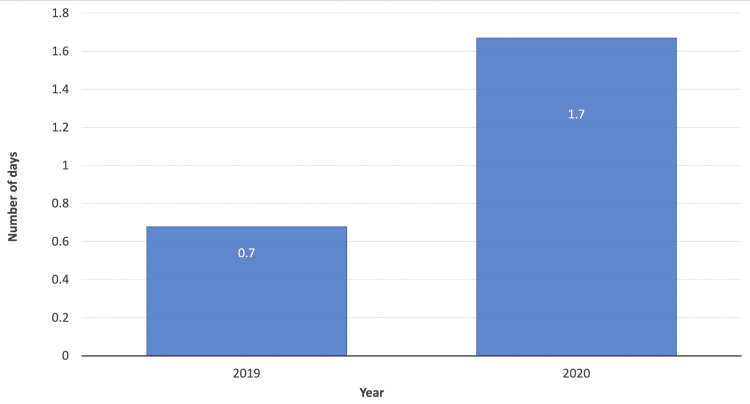
Average number of days from injury to presentation in March-June 2019 and 2020

**Figure 3:**
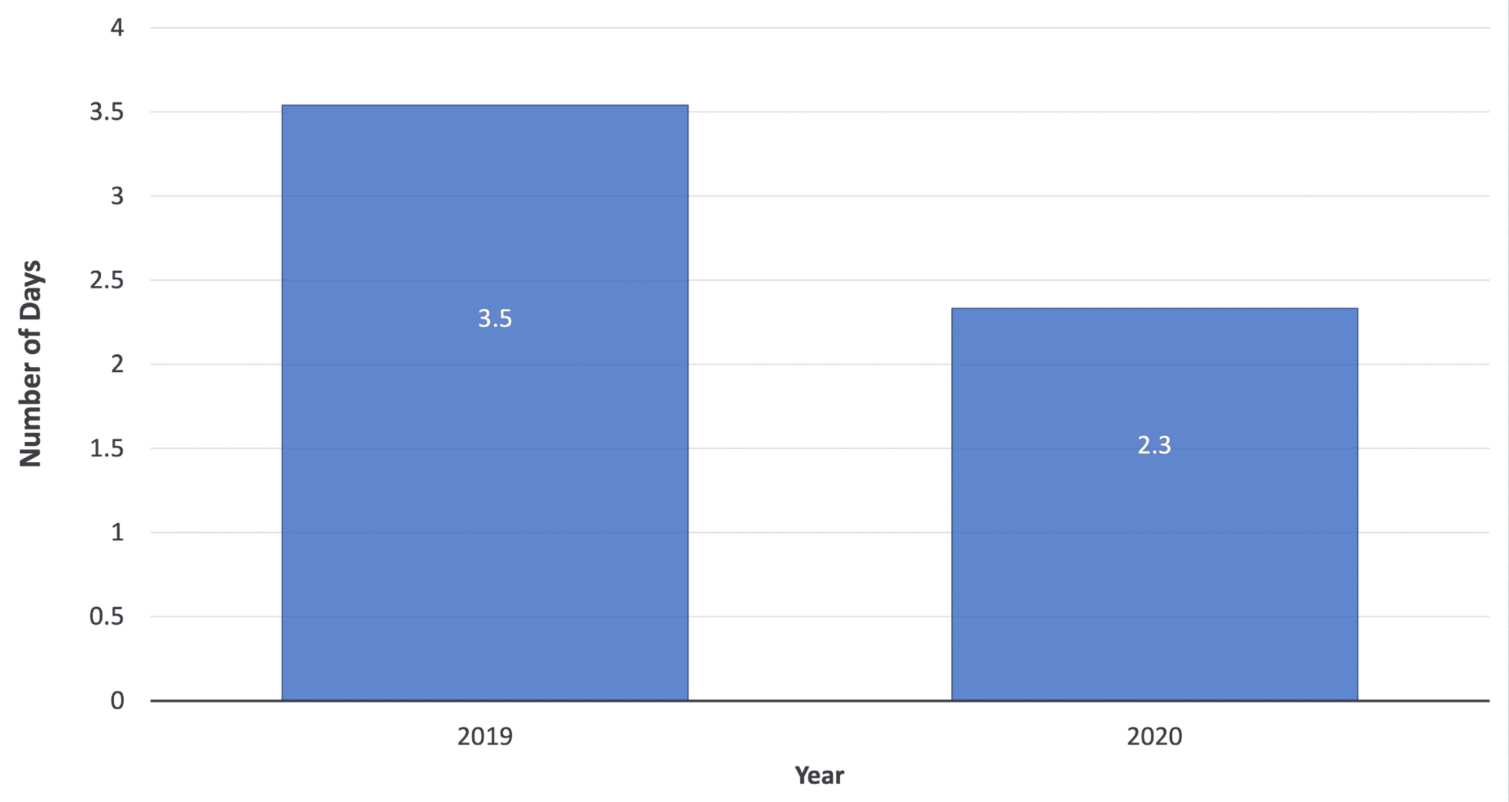
Average number of days from presentation to operation in March-June 2019 and 2020

​The graph in Figure 2 depicts the average number of days from injury to presentation at the orthopaedic department. In March-June 2019, the average number of days from injury to presentation was 0.7. This significantly increased to 1.7 days in March-June 2020 (p=0.009). The data in Figure 3 also shows that the average number of days from presentation to surgery was 3.8 days in March-June 2019. This significantly decreased to an average of 2.5 days in March-June 2020 (p=0.020).

Mechanism of injury

**Figure 4:**
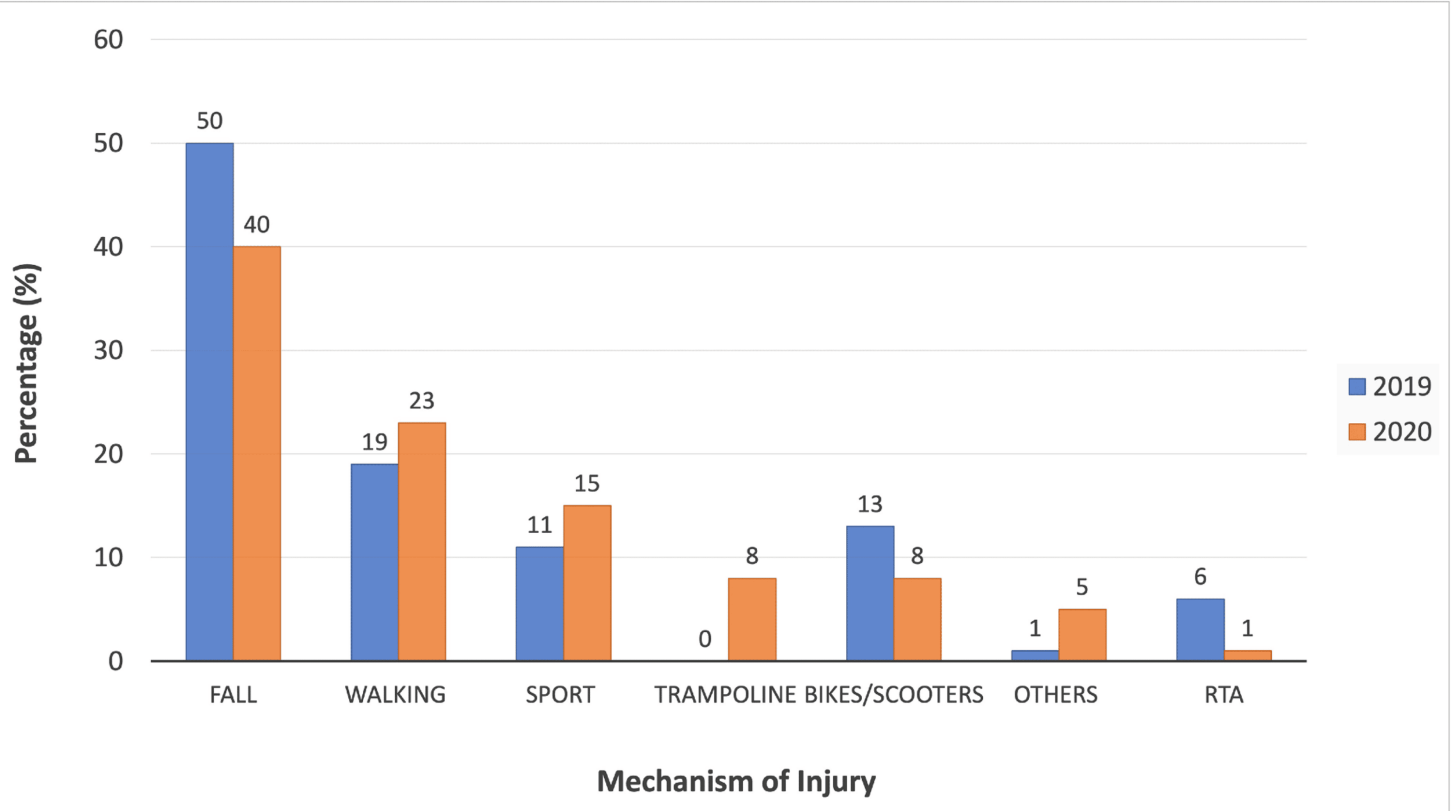
Percentage change in mechanism of injury for ankle fractures in March-June 2019 and 2020

​As shown in Figure 4, the most frequent mechanism of injury leading to acute ankle fractures was attributed to low-energy falls, with 50% of cases (n=45) from March to June 2019 having sustained an ankle fracture due to a fall. However, from March to June 2020, low-energy falls attributed to 40% (n=65). The graph in Figure 4 also illustrates that walking and sports were mechanisms that contributed towards an increased number of acute ankle fractures in March-June 2020 when compared to 2019. Trampoline accidents were responsible for 8% (n=12) of ankle fractures in March-June 2020; however, there were no trampoline-related ankle injuries reported in March-June 2019. Furthermore, road traffic accidents (RTAs) decreased from 6% (n=5) in March-June 2019 to 1% (n=2) in 2020. 

Patient outcomes

​Data were analysed to assess for differences between patient outcomes, measured using the Foot and Ankle Outcome Score (FAOS), and several variables of interest. FAOS scores were significantly higher in 2020 compared to 2019 (mean difference (MD): -10.29, standard deviation (SD): -22.31 to 1.94, p=0.049). No statistically significant difference was found between sex and FAOS score (MD: 10.24, SD: -2.877 to 23.35, p=0.062 (independent samples t-test)). Similarly, the data showed no significant difference between the FAOS score and the number of days from injury to surgery (Pearson correlation coefficient: -0.246, p=0.131).

## Discussion

​The current state of the COVID-19 pandemic has forced a review of hospital services, with the British Orthopaedic Association Standards in Trauma and Orthopaedics (BOAST) introducing new COVID-19-related guidelines [4]. The principal strategy employed by this NHS trust during the height of the pandemic was to minimise patient admissions and ease the use of limited healthcare resources in anticipation of a surge of COVID-19 patients. Consequently, the NHS trusts were forced to adapt to meet new demands and prioritise patients based on clinical needs. Adaptations included operating theatres being converted to host critical care beds [5].

​Interestingly, Wong et al. found that during the peak of the pandemic, the ratio of orthopaedic trauma operations to elective operations more than doubled, when compared with figures published one year prior [6]. This interesting finding also resonates with our study, as our orthopaedic elective cases were cancelled in a conscious attempt to keep non-emergency cases at home and protect them from potentially contracting a nosocomial-associated infection, notably COVID-19. However, from March to June 2020, there was an evident increase in acute ankle cases. A total of 161 ankle fractures were seen in our study period in 2020 compared with only 90 cases one year prior (Table 1). ​This study was set over a four-month period, with 72% of ankle fractures being managed conservatively, a huge contrast to that of 2019, where only 20% of cases were managed conservatively (Figure 1). Interestingly, a recent study by Park et al. also had corroborating results [7].

​The significant differences in management demonstrate the attempts the department took to protect patients and staff from potential COVID-19 exposure and minimise utilisation of limited hospital resources, which resulted in fewer cases undergoing surgery and an increased number being managed conservatively and away from the hospital. Despite this, our data demonstrates that the functional outcome (FAOS scores) was non-inferior in the predominantly conservatively managed cohort (March-June 2020) compared to the pre-pandemic cohort (MD: -10.29, SD: -22.31 to 1.94, p=0.049). This greater proportion of conservatively managed ankle fractures in our orthopaedic department was in line with COVID BOAST 12 guidelines and in line with the UK Ankle Injury Management (AIM) trial [4,8]. This trial of 620 ankle fractures showed equivocal outcomes at six months when comparing conservative management (casting) with open reduction internal fixation (ORIF) but at reduced costs [8]. A recent study by Yap et al. (2020) discussing the functional outcome of operative versus non-operative management of ankle fractures also demonstrated that ankles managed conservatively had satisfactory and similar outcomes as patients managed surgically [9]. However, those that were managed conservatively had stable fractures with joint congruity and no obvious medial malleolus disruption [9].

​Similarly, Javed et al. also found that surgical and conservative management of unstable ankle fractures during the COVID-19 period produced similar short-term functional outcomes [10]. However, this study also identified that ankle fractures managed conservatively were at a higher risk of treatment failure and malunion/non-union when compared with those managed surgically [10].

​The rise in ankle fracture case numbers during the COVID-19 lockdown phase may be attributed to restricted social mobility in combination with a lack of health and fitness establishments resulting in domestic restlessness and eventuating in the rise of medium energy injuries indoors (Figure 2). Interestingly, a study by Ding et al. showed that the level of interest in physical exercise during April 2020 from the general population of the UK, USA and Australia demonstrated an upward trend [11]. ASA classification determines the extent of disease and limitation on activities, and this reported increase in interest in physical activity following the COVID-19 lockdown may account for the non-statistically significant difference in pre-fracture ASA grading between our two cohorts (p=0.409).

​Furthermore, our study found that simple falls at home contributed towards the vast majority of ankle fractures during the COVID-19 lockdown phase. In addition, trampoline accidents contributed to 8% of ankle fractures during the 2020 COVID-19 lockdown phase, a stark contrast to 2019’s data, which did not attribute any ankle injuries to this mechanism. This could be due to trampolining being an inexpensive and accessible form of home exercise. A similar increase in trampoline injuries was also noted in a study of paediatric trauma in Broomfield’s Hospital, Essex, during the 2020 COVID-19 lockdown months [12]. The study by Staunton et al. (2020) investigated how orthopaedic trauma cases had been affected as a result of the lockdown [13]. It also concluded that there was a significant reduction in sport-related injuries, suggestive of compliance with COVID-19 activity guidelines. Nevertheless, there was an increase in injuries associated with home personal exercises and simple falls at home [13]. It is of note that ankle fractures due to road traffic accidents (RTAs) had actually decreased from 6% in March-June 2019 to 1% in 2020 (Figure 2). This reflected similar findings in a study by Qureshi et al., who noted that there was also a decreased number of RTA-associated injuries during the COVID-19 pandemic of 2020 [14]. This again can be explained by the general public abiding with COVID-19 lockdown rules, staying at home and not travelling unless absolutely necessary.

​There was a significant change with regard to delay in injury to presentation when assessing March-June 2019 and 2020 data (p=0.009). It took on average 0.7 days for a patient to present to the orthopaedic department in non-COVID-19 times (March-June 2019), but there was an increase in delay to presentation during COVID-19 times, with the average day to presentation amounting to 1.7 days. Lynn et al. identified that during the pandemic, there was an increase of 32% in delay of injury to presentation when compared to the non-pandemic period, with a possible explanation being patients having apprehensions about attending a hospital due to the risk of contracting the COVID-19 virus [15].

​Moreover, the delay from presentation to surgery showed a significant difference (p=0.020) when the two study periods were compared. The delay from presentation to surgery in non-COVID-19 times was 3.5 days compared with 2.3 days during the COVID-19 pandemic. This reduction in delay to surgery could be attributed to a multitude of reasons such as reduced overall surgical referrals from accident and emergency, reduced number of elective cases and resultantly a larger availability of surgeons to operate, with only theatre time and operating space as limiting factors [5,7].

​There are several strengths to this study. Firstly, this study encapsulates a large sample size (N=251) across a total of six months, which increases the internal validity of our data. In addition, using data from the same department standardises the operating surgeons as well as the demographic spread of the patient population. Furthermore, the data acquired from the secure electronic hospital database was cross-checked with physical notes to reduce information bias. Nevertheless, there are some notable limitations. As this is a case-control study, not all confounding variables of our study population were considered. Dates were chosen to minimise the seasonal variance of orthopaedic referrals and maximise inclusion of patients affected by the COVID-19 lockdown, and due to the strict case definition being defined a priori, the study population could be subject to a degree of selection bias. Finally, as this is a single-centre study, the external validity and generalisability of the data are limited.

## Conclusions

​Compared to the pre-COVID-19 pandemic period, more ankle fractures were managed conservatively in 2020, despite no significant difference found in baseline morbidity. In addition, patient outcomes were greater in 2020. Going forward, having a higher threshold for surgical intervention for ankle fractures appears prudent in light of the latest BOAST guidance and our data demonstrating the non-inferiority in patient outcomes when conservatively managed.

## Figures and Tables

**Figure 5:**
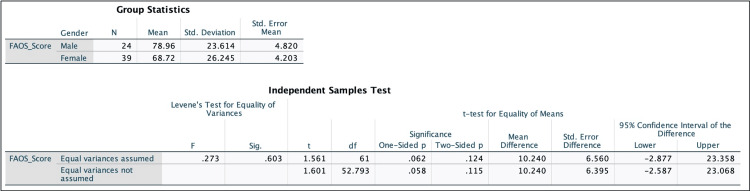
Patient outcomes (by FAOS score) between 2019 and 2020 Equal variances not assumed due to positive Levene’s test (<0.05) Conclusion: MD: -10.187, p=0.049 (-22.314 to 1.941) FAOS: Foot and Ankle Outcome Score, MD: mean difference

**Figure 6:**
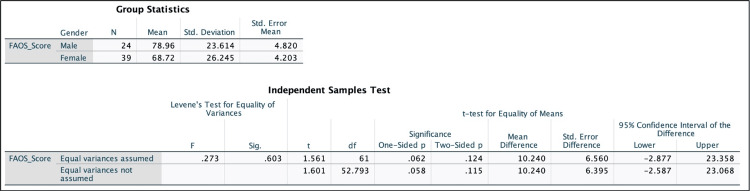
Relationship between sex and patient outcomes (FAOS) Equal variances assumed due to negative Levene’s test (>0.05) Conclusion: MD: 10.24, p=0.062 (-2.877 to 23.35) FAOS: Foot and Ankle Outcome Score, MD: mean difference

**Figure 7:**
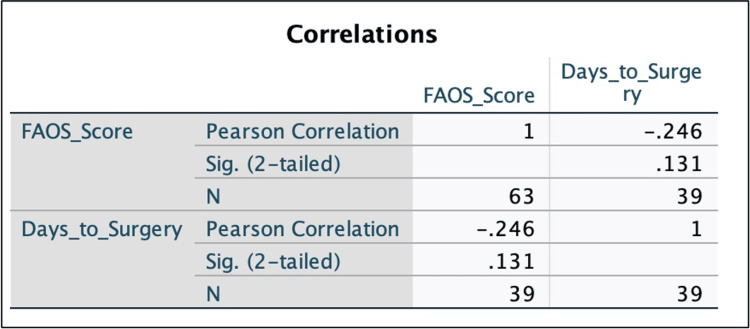
Relationship between days to surgery and patient outcomes (FAOS) Conclusion: Pearson coefficient: -0.246 (low negative correlation), p=0.131 FAOS: Foot and Ankle Outcome Score

**Figure 8:**
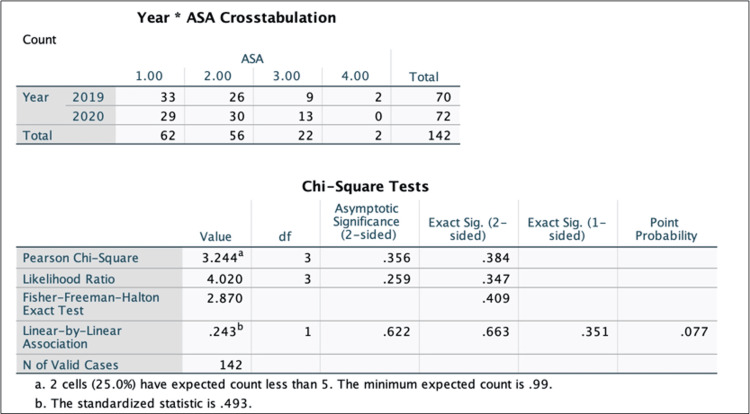
Difference in ASA grades between 2019 and 2020 Assumption violated, 25.0% (>20%) Exact method: Fisher’s exact test value: 2.817, p=0.409

## Appendices

Figure 5 shows patient outcomes (by FAOS score) between 2019 and 2020.

Figure 6 shows the relationship between sex and patient outcomes (FAOS).

Figure 7 shows the relationship between days to surgery and patient outcome (FAOS).

Figure 8 shows the difference in ASA grades between 2019 and 2020. 
